# Tackling the social cognition paradox through multi-scale approaches

**DOI:** 10.3389/fpsyg.2014.00882

**Published:** 2014-08-12

**Authors:** Guillaume Dumas, J. A. Scott Kelso, Jacqueline Nadel

**Affiliations:** ^1^Human Brain and Behavior Laboratory, Center for Complex Systems and Brain Sciences, FAUBoca Raton, FL, USA; ^2^Intelligent System Research Centre, University of UlsterDerry, Northern Ireland; ^3^CRICM UMR-S975, UPMC-Paris 6Paris, France; ^4^CNRS, UMR 7225Paris, France; ^5^ICMParis, France

**Keywords:** multiscale, social interaction, developmental psychology, two-body neuroscience, second person perspective, hyperscanning, coordination dynamics, complex systems

Recent debates regarding the primacy of social interaction versus individual cognition appear to be caused by the lack of an integrative account of the multiple scales at play. We suggest that reconciling individual autonomy and dyadic interactive viewpoints requires the taking into account of different time scales (e.g., development, learning) and levels of organization (e.g., genetic, neural, behavioral, social). We argue that this challenge requires the joint development of tools for two-body and second person neuroscience, along with the theoretical concepts and methods of coordination dynamics and systems biology. Such a research program may be particularly fruitful in deciphering complex socio-developmental diseases that are known to involve alterations on multiple levels.

## The ontogeny of social cognition: a chicken-egg issue?

Despite a propensity to interact with others, our ability to socialize seems neither given nor fixed once and for all (Dumas, 2011). As Sheets-Johnstone (2011) has pointed out “we come into the world moving; we are precisely not stillborn.” The question of the ontogeny of social cognition (mirror neurons included) is grounded in our propensity to move. This primacy of movement can even be observed before birth: motorneurons appear well before their sensory counterparts in embryo; a large repertoire of spontaneous (thus self-organized) movements—e.g., making a fist, kicking, sucking—already exists (Kelso, 2002; Piontelli, 2010). Even twin fetuses demonstrate distinctive movements directed to each other (Castiello et al., 2010). At this stage, the “social events” are essentially movements. Does this mean, however, that there is no element of “social cognition” in such encounters? We think not.

Behavioral coordination acts as a powerful linkage between persons, even early in life. Infants are sensitive to contingent movements of the mother (Nadel et al., 1999) and the first dyadic interactions already exhibit co-regulation, “a continuous mutual adjustment of actions and intentions” (Fogel and Garvey, 2007). The disposition of human and monkey newborns to imitate (Meltzoff and Moore, 1983; Kugiumutzakis, 1993; Nagy et al., 2005; Ferrari et al., 2006; Soussignan et al., 2011) is not due to a passive coupling of perception and action. Rather it is an active attempt to adapt and gradually refine their own movements with respect to others. When imitated, human infants and newborn macaques display affiliative behavior toward the imitator (Paukner et al., 2009), as do low-functioning children with Autism Spectrum Disorder (ASD) (Nadel et al., 2000). The two facets of imitation, imitate and be imitated, constitute dual roles that can be traded, thereby allowing turn-taking (Nadel-Brulfert and Baudonnière, 1982). All that is needed is anticipation of the partner's next movement.

Here it seems we arrive at a cross-road: key ingredients of social cognition already appear to be present very early. Co-regulation of synchrony, anticipation of the other's intentions, joint attention on a physical target, are central facets of social interaction. Does this mean they all emerge from the developing Mirror Neuron System (MNS)? Even if the early capacity to couple perception and action is associated with a proto MNS (Lepage and Théoret, 2007), we appear to be confronted with a circular logic problem: you need a MNS for social interaction but you need to interact to form a MNS. Although there is limited evidence for mirror neurons in early development (Catmur, 2013), sensorimotor experience may indeed be key to creating mirror neuron responses through Hebbian learning (Keysers and Perrett, 2004; see also Allen and Williams, 2011). See also the epigenetic view of Ferrari et al. (2013).

The idea that the MNS underlies not only motor exchanges but also high-level social cognition is now challenged by the proposal of a complementary role for the “mentalizing network” (Keysers and Gazzola, 2007; Uddin et al., 2007; Sperduti et al., 2014). A main task is to decipher possible top-down and bottom-up processes in social cognition. Such an endeavor requires, at the very least, joint investigation of behavioral and neural dynamics during real social exchanges (Hari and Kujala, 2009; Schilbach, 2014).

## The rise of two-body and second-person neuroscience

Although social neuroscience has gathered a lot of data on how individual human beings perceive social stimuli, a truly interactive social neuroscience still lags behind. The community seems to have reached a consensus on the importance of investigating social situations that involve reciprocal exchange and mutual engagement (Hari and Kujala, 2009; Schilbach et al., 2013). Technological developments such as hyperscanning (Tognoli et al., 2007; Dumas et al., 2010; Babiloni and Astolfi, 2012; Hasson et al., 2012; Konvalinka and Roepstorff, 2012) and human-machine interfaces (Kelso et al., 2009; Pfeiffer et al., 2011) have greatly helped operationalize various aspects of real-time social interaction, thereby narrowing the gap between what we know about off-line and on-line social cognition (Schilbach, 2014). The former not only involve the same brain structures identified in research on isolated individuals (Sperduti et al., 2014); the brain dynamics vary according to social context, e.g., spontaneous vs. instructed interaction (Dumas et al., 2012a; Guionnet et al., 2012; Sänger et al., 2012) and social role, e.g., leaders vs. followers (Dumas et al., 2012a; Sänger et al., 2013; Konvalinka et al., 2014).

A further challenge concerns the structure and timing of inter-individual coordination and its relationship with intra-individual processes. Functional magnetic resonance imaging (fMRI) hyperscanning first showed strong anatomical and functional similarities across different individuals responding to the same perception, especially if it is social (Hasson et al., 2004). This finding extends to interactive contexts where inter-brain synchronization emerges in multiple frequency bands (Dumas et al., 2010; Müller et al., 2013). The related symmetrical and asymmetrical inter-brain patterns reflect how social interaction goes beyond a simple mirroring of the other and relies both on grasping other individuals' motor goals and inferring their intentions (Nadel and Dumas, 2014). Moreover, unlike intra-brain dynamics which primarily involves high frequency rhythms, the inter-brain dynamics appear to operate at lower frequencies (Müller et al., 2013). Thus, the temporal interplay between brain networks involved in social interaction, such as the so-called mirror and mentalizing systems, may be modulated by dynamics at the dyadic level, as in turn-taking (Wilson and Wilson, 2005). Moreover, social cognition cannot be understood only on the bases of intra- or inter-personal dynamics but rather in their common hyper-brain space including both intra- and inter-brain coupling dynamics (e.g., Montague et al., 2002; De Vico Fallani et al., 2010; Sänger et al., 2012, 2013; Müller et al., 2013).

## Social dynamics as a bridge between scales

Cognition is constantly evolving during interactions with the environment and others. In order to sustain covariation, members of a social interaction must engage in active co-regulation (Fogel, 1993) and co-anticipation (Nadel and Dumas, 2014), potentially leading to the co-ownership of the action (Dumas et al., 2012a). Such genuine sharing of the interaction with others has been proposed as participatory sense-making (De Jaegher and Di Paolo, 2007) where social interaction plays a constitutive role for individual cognition (De Jaegher, 2009; Froese et al., 2014). The chicken-egg paradox here vanishes since both interactive and non-interactive mechanisms co-develop and mutually shape each other's development (Di Paolo and De Jaegher, 2012). Although still debated (Gallotti and Frith, 2013), this proposal is now supported by both modeling (Froese and Di Paolo, 2010; Froese et al., 2013) and experimental research (Auvray et al., 2009; Froese et al., 2014). In studies that have assessed the emergence of collective intelligence through dialog (Bahrami et al., 2010; Bang et al., 2014) interaction has been shown to constrain individual information processing (Fusaroli et al., 2014).

Social cognition thus relies on a braiding of neural, behavioral, and social processes (Hari and Kujala, 2009; Kelso et al., 2013). Neurobiological models of socio-cognitive functions have already been proposed (Gallese et al., 2004; Keysers and Perrett, 2004; Friston et al., 2011), though the dynamical components of human interaction are still largely missing (Adolphs, 2003). The theoretical and empirical framework of coordination dynamics has shown that neural, behavioral, and social scales may be studied and understood from a common perspective (Kelso, 1995; Kelso et al., 2009, 2013). As in other theories that aim to elaborate mathematical formalisms for cognition (e.g., Tononi, 2008; Friston, 2010), the objective of coordination dynamics is to identify general principles, the mechanistic realizations of which may be found in a variety of different systems at multiple levels of description. To be more than just words, coordination dynamics had to establish experimentally that criterial features of self-organization (e.g., order parameters, control parameters, stability, instability) actually existed in human behavior and that they could be mapped explicitly on to a theoretical model of the self-organizing dynamics. Then it had to show how information (e.g., about goals, intentions, the environment, etc.) shapes and is shaped by the self-organizing dynamics. Coordination dynamics relies on the same concepts and mathematical formalisms across different time scales and organizational levels and thus potentially offers inroads into a multi-scale account of social cognition.

In physics, multi-scale approaches have already uncovered universal principles, especially when matter undergoes phase transitions (Wilson, 1979). At the neural level, non-linear cross-scale interactions have been demonstrated experimentally (Le Van Quyen, 2011; see also Plenz and Niebur, 2014). In social neuroscience, nonlinearities are omnipresent in the underlying neural and social dynamics. Since functional networks display similar behavior across time-scales (Kelso, 1995; Bressler and Tognoli, 2006), a parsimonious account may be possible. Beyond the quest for parsimony and semantic clarity, having a mathematical formalism enables one to ask computationally relevant questions. For example, in the case of social neuroscience, neurocomputational modeling shows that the anatomical structure of the human brain favors both the complexity of intra-individual dynamics and the coupling in inter-individual dynamics (Dumas et al., 2012b). Regarding the debate about the constitutive role of social interaction, future computational studies can quantify macro-to-micro causal effects ranging from dyadic to individual processes (Hoel et al., 2013).

## Conclusion

Social interaction challenges the boundaries between the field of cognitive science and how to divide observations across distinct time scales and organizational levels. Social neuroscience is taking up this challenge at both theoretical and methodological levels. Here we have argued that three major dimensions are of potential significance: integrating a developmental perspective, investigating real-time social interaction with a two-body or second person neuroscience, and adopting a multi-scale approach through complex systems' perspectives, in particular the concepts, methods and tools of coordination dynamics. These developments have already begun and should help further an understanding of disorders of social interaction such as autism.

As Abney et al. (2014) have remarked, in cognitive science “multiple theories should interact when describing the same phenomenon.” In social cognition, the case of autism provides a test bed for an integrative approach. Developmental psychopathology has uncovered a wide range of behavioral peculiarities of persons with autism (Burack et al., 2002); cognitive neuroscience has identified many biomarkers at both structural and functional levels; and systems biology has begun to relate genetic variants associated with cellular and metabolic pathways to individual behavior (Randolph-Gips, 2011). The next logical step is to bridge the gap between multiple levels (and disciplines). Two-body or second-person approaches have already drawn some connections between neural and social dynamics in neurotypical populations, and provide potentially powerful tools for the investigation of autism. Hyperscanning techniques, for instance, can be used to uncover relationships between phenotypes at the behavioral level and endophenotypes at neural levels. Inter-individual computational models combined with hyperscanning data could help elucidate causal relationships between structure and dynamics. Differences in brain anatomy may impact the ability of persons with autism to couple with others early in life thus decreasing their propensity to develop social skills (Dumas et al., 2012b). Computational neurogenetic approaches can help model the relationship between the genetics of autism and brain dynamics (Benuskova and Kasabov, 2008). Such integration of neurogenetics and systems biology may soon aid in tackling the heterogeneity observed in autism across genotype, neural endophenotype, and socio-behavioral phenotype levels.

### Conflict of interest statement

The authors declare that the research was conducted in the absence of any commercial or financial relationships that could be construed as a potential conflict of interest.
